# Whole-pelvic irradiation with boost to involved nodes and prostate in node-positive prostate cancer—long-term data from the prospective PLATIN-2 trial

**DOI:** 10.1007/s00066-023-02129-y

**Published:** 2023-08-28

**Authors:** C. A. Fink, D. Wegener, L. D. Sauer, C. Jäkel, D. Zips, J. Debus, K. Herfarth, S. A. Koerber

**Affiliations:** 1 Department of Radiation Oncology, INF 400, Heidelberg University Hospital, 69120 Heidelberg, Germany; 2 Heidelberg Institute for Radiooncology (HIRO), INF 400, National Center for Radiation Research in Oncology (NCRO), 69120 Heidelberg, Germany; 3 INF 460, National Center for Tumor Diseases (NCT), 69120 Heidelberg, Germany; 4 Department of Radiation Oncology, University Hospital Tuebingen, Hoppe-Seyler-Straße 3, 72076 Tuebingen, Germany; 5 University of Heidelberg, INF 130.3, Institute of Medical Biometry (IMBI), 69120 Heidelberg, Germany; 6 Clinical Cooperation Unit, INF 280, German Cancer Research Center (DKFZ), 69120 Heidelberg, Germany; 7 INF 450, Heidelberg Ion Beam Therapy Center (HIT), 69120 Heidelberg, Germany; 8 Department of Radiation Oncology, Barmherzige Brueder Hospital Regensburg, Pruefeninger Straße 86, 93049 Regensburg, Germany

**Keywords:** Regional and distant metastasis, Radiotherapy, Pelvic lymph node, Toxicity, Quality of life

## Abstract

**Purpose:**

Node-positive prostate cancer is a potentially curable disease. Definitive radiotherapy to the prostate and lymphatic drainage is an effective treatment option but prospective long-term outcome data are scarce. Thus, the current study aimed to evaluate the toxicity and efficacy of definitive radiation therapy for men with prostate cancer and nodal metastases using modern irradiation techniques.

**Methods:**

A total of 40 treatment-naïve men with node-positive prostate cancer were allocated to the trial. All patients received definitive radiation therapy at two German university hospitals between 2009 and 2018. Radiation was delivered as intensity-modulated radiation therapy (IMRT) with 51 Gy to the lymphatic drainage with simultaneous integrated boost (SIB) up to 61.2 Gy to involved nodes and 76.5 Gy to the prostate in 34 fractions. Feasibility and safety, overall and progression-free survival, toxicity, and quality of life measurements were analyzed.

**Results:**

During a median follow-up of 79 months, median overall survival was 107 months and progression-free survival was 78 months. Based on imaging follow-up, no infield relapse was reported during the first 24 months of follow-up. There were 3 (8%) potentially treatment-related grade 3 toxicities. Common iliac node involvement was associated with a higher risk of progression (HR 15.8; 95% CI 2.1–119.8; *p* = 0.007).

**Conclusion:**

Definitive radiation to the lymphatic drainage with SIB to the involved nodes and prostate is a safe and effective treatment approach for patients with treatment-naïve, node-positive prostate cancer with excellent infield tumor control rates and tolerable toxicity. Location rather than number of involved nodes is a major risk factor for progression.

**Supplementary Information:**

The online version of this article (10.1007/s00066-023-02129-y) contains supplementary material, which is available to authorized users.

## Introduction

With an incidence of 130 per 100,000 males per year, prostate cancer is the most common newly diagnosed noncutaneous cancer in European men [1]. In case of node-positive prostate cancer at primary diagnosis, the mainstay of treatment is either radical prostatectomy with pelvic lymph node dissection or definitive radiation to the prostate and pelvic nodes both combined with androgen-deprivation therapy [2]. As safe and effective dose concepts have been established for normal fractionation, hypofractionation, and ultrahypofractionation for the prostate over the years [3, 4], fewer long-term outcome data have been published on toxicity and efficacy of dose concepts with the inclusion of positive nodes and elective pelvic lymphatic drainage. Due to the lack of comparative trials and supported by higher-sensitivity diagnostics such as PSMA-PET/CT, a multitude of surgical techniques and radiation approaches are currently used to treat primary node-positive prostate cancer. To minimize toxicity, some techniques aim for involved-node therapy (i.e., lymph node SBRT, PSMA-guided surgery), with a risk of leaving potential disease in the pelvis untreated.

The five-armed, prospective PLATIN phase II trials aim at determining safety and feasibility, progression-free survival, and toxicity of IMRT of the pelvic nodes in patients with a SIB to either the prostate (PLATIN‑1 [5, 6]) or the prostate and macroscopic nodes (PLATIN-2) in patients with a high risk for (Roach formula > 20%) or clinical suspicion of nodal involvement. In a postoperative setting, PLATIN investigates pelvic node irradiation with a SIB to the prostate bed (PLATIN‑3 [7]), the prostate bed and involved nodes (PLATIN-4), or to involved nodes in patients with nodal recurrence after having received previous adjuvant prostate bed irradiation (PLATIN-5). Here, we report on the long-term outcome data of the PLATIN-2 trial.

## Methods

### Study design and participants

PLATIN‑2 is an investigator-initiated, prospective, open, bicentric, phase II trial initiated in 2009. Between 2009 and 2018, 40 patients with biopsy-proven, treatment-naïve prostate cancer and clinical suspicion of node-positive disease in the pelvis at primary diagnosis were enrolled in the trial. Eligible patients were between the age of 18 and 75 and had a sufficient performance status (Karnofsky performance status ≥70%). The study protocol required androgen-deprivation therapy (ADT) for at least 2 months prior to study entry as well as ADT covering the period of radiation therapy (usually 3-month depot LHRH-analogue). A total ADT period of 24 months was recommended. Exclusion criteria included pretherapeutic lymph edema, previous radiation to the pelvis, and metastatic disease outside of the pelvis.

All patients had pretherapeutic staging with conventional imaging (pelvic CT and MRI and bone scintigraphy), ^18^F‑fluorethylcholine, or [^68^Ga]Ga-PSMA-PET/CT. Prostate-specific antigen (PSA) values were documented before the start of ADT, before radiation, and during follow-up visits. Follow-up visits were performed 6 weeks and 6, 12, 18, and 24 months after completion of radiotherapy and PSA values were documented every 3 months. For post-hoc analysis, overall survival (OS) was enquired with the German Cancer Registry. Patients were contacted for quality of life measures and PSA history was provided by the treating urologist.

### Procedures

Full details on the radiation parameters are provided in the protocol. In summary, radiation was delivered as external beam radiation therapy either as step-and-shoot IMRT or tomotherapy, both with daily image guidance. No gold fiducials were implanted. Target volume matching was primarily performed to the prostate with a PTV margin of 6 mm and secondarily to the lymphatic drainage and metastatic nodes, each with a PTV margin of 5 mm. All patients received irradiation of the pelvic lymphatic drainage with a total dose of 51 Gy with a SIB up to 76.5 Gy to the prostate and 61.2 Gy to lymph nodes suspicious of metastatic disease in 34 fractions. To minimize toxicity, the boost to the involved nodes was considered secondary in close proximity to the intestinal wall, permitting a maximum dose of 53 Gy in the intestine. Irradiated elective lymphatic drainage covered the external and internal iliac, obturator, presacral, and common iliac nodes. Patients were offered therapy response assessment with MRI 6, 12, 18, and 24 months after therapy completion. With regard to nodal disease, therapy response assessment was conducted according to the revised RECIST guidelines (version 1.1) [8].

### Outcomes

Due to the study protocol being designed in 2009, the primary endpoint of the PLATIN trial was safety and feasibility of the IMRT/IGRT technique in patients with prostate cancer and an indication for nodal irradiation with a SIB to lymph node metastases. Safety and feasibility were assessed based on the rate of grade 3–5 toxicities (EORTC CTCAE v3.0) and number of treatment discontinuations, respectively.

One secondary endpoint was progression-free survival (PFS), defined as time from study entry to biochemical failure, radiological disease progression, or death from any cause. Biochemical failure was defined according to the Phoenix recommendations [9]. Radiological progression was determined by pelvic MRI during the 6‑, 12-, 18-, and 24-month follow-up visits. If MRI was contraindicated for staging due to comorbidities, contrast-enhanced pelvic CT scan was performed. OS was defined as death from any cause. Additionally, quality of life was determined by EORTC QLQ-C30 questionnaires and analyzed in accordance with EORTC guidelines [8].

The safe treatment application rate (STR) was chosen as the primary endpoint and defined as the proportion of patients without treatment discontinuation or grade 3–5 toxicity as reported before [5].

The secondary survival endpoints were analyzed using the following methods: a Kaplan–Meier plot was generated in order to describe the development of OS and PFS over time. Furthermore, to assess the effect of different covariates on PFS, a multivariate Cox regression including all covariates of interest was performed. The proportional hazards assumption necessary for correctly applying the Cox regression was checked using a score test and graphical methods.

The secondary quality of life endpoints were analyzed depicting the development of population mean and standard error over the first six visits (baseline and months 2, 3, 6, 12, and 24) in a line plot with error bars.

The figures were created with RStudio (2022.02.3 + 492, packages “survminer,” “ggplot2,” “ggpubr,” “forester”) [10].

The PLATIN protocol is conducted in accordance with Good Clinical Practice guidelines and the Declaration of Helsinki. Ethics approval was granted by the local ethics committee of both participating centers.

## Results

Between 2009 and 2018, 40 patients with pelvic node-positive prostate cancer at primary diagnosis were allocated to the PLATIN-2 trial for definite radiation therapy. The baseline characteristics of the patients are shown in Table 1. The median age at study entry was 69.5 years (range 53–75), median PSA was 26.5 ng/ml (range 6.6–232), and all patients had intermediate- or high-risk-featured biopsy-proven primary tumors. Suspicion for nodal metastases was based on conventional imaging (pelvic CT/MRI) alone in 24/40 (60%) and on PET/CT in 16/40 (40%) of men. All patients had either PET/CT and/or bone scintigraphy negative for metastatic bone lesions. A SIB was applied to a median of 2 (range 1–8) lymph node metastases with a nodal boost planning target volume of 17 ml (range 3.1–107). ADT was started 2 months prior to radiation therapy with a PSA reduction proving hormone sensitivity in all cases. All patients reported a minimum of 6 months ADT. Although 24 months of ADT were recommended, 29/40 (72.5%) had a documented androgen suppression for 18–30 months, 6/40 (15%) discontinued the ADT after 6 months, and for 5/40 patients (12.5%), no reliable, continuous data for ADT use were documented after 6 months of follow-up.

**Table 1:** Patient and planning characteristics

*Patients (n)*	40
*Age at study entry (years)*	
Median (range)	69.5 (53–75)
*PSA before ADT at study entry (ng/ml)*	
Median (range)	26.5 (6.6–232)
*Gleason score (n)*	
6	1 (3%)
7a	6 (15%)
7b	10 (25%)
8	14 (35%)
9	8 (20%)
10	1 (3%)
*T stage at primary diagnosis*	
T1	13 (33%)
T2	6 (15%)
T3	16 (40%)
T4	5 (13%)
*Pretherapeutic staging (n)*	
Conventional only	24 (60%)
Choline PET/CT	12 (30%)
Choline and PSMA-PET/CT	2 (5%)
PSMA-PET/CT	2 (5%)
*Boosted lymph nodes per patient (n)*	
Median (range)	2 (1–8)
*PTV of total LN-SIB (cm*^*3*^*)*	
Median (range)	17 (3.1–107)

**Fig. 1:**
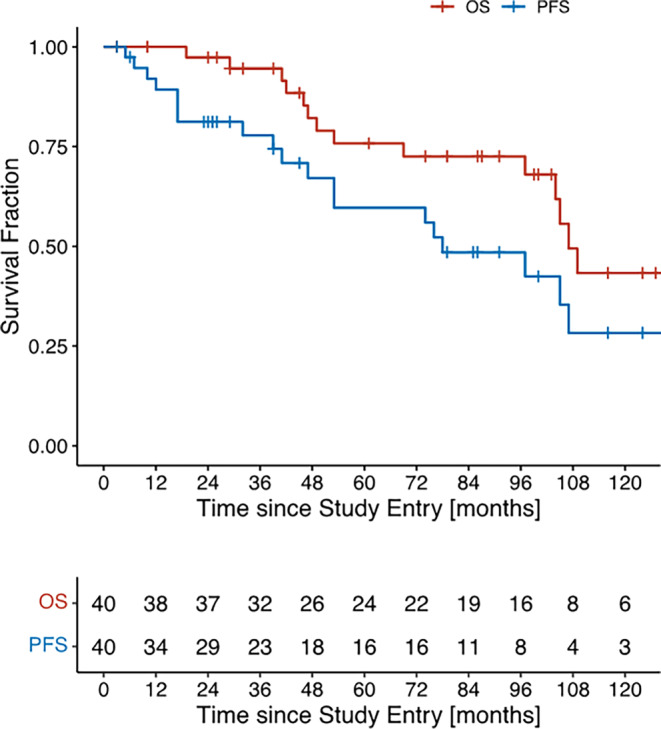
Survival data PLATIN‑2. *OS* overall survival, *PFS* progression-free survival

The median follow-up period regarding OS data was 79 months (range 3–141) with a median OS of 107 months (Fig. 1). PSA values were available for a median of 49 months (range 3–141) with a PFS of 78 months.

In 104 posttherapeutic abdominal MRI or CT scans in 31 patients, no infield relapse was seen up to 24 months after study entry. Based on RECIST 1.1 criteria, 61% (19/31) of patients had partial or complete remission after 24 months; 16% (5/31) were diagnosed with progressive disease on follow-up MRI during the first 2 years of follow-up. All relapse sites were located out of the radiation field and occurred in patients with initially conventional staging at an early follow-up stage. At long-term follow-up after a median of 49 months, 14/40 (35%) patients had experienced disease relapse. Relapse pattern analysis revealed an outfield progression in 11 patients and one patient with both outfield and infield tracer avidity shown on PSMA-PET/CT. In two patients, no imaging data were available after biochemical relapse.

**Fig. 2:**
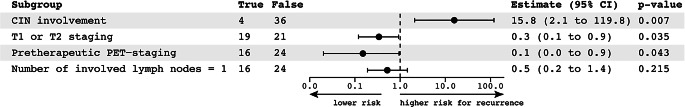
Hazard risk for progression in subgroups. *CIN* common iliac node, *PET* positron-emission tomography

In multivariate analysis, a lower risk of progression was seen in patients with only one involved lymph node and T1/T2 disease (Fig. 2). Regarding the primary staging before study entry, the risk of progression was higher in patients with conventional staging compared to patients staged with PET/CT (*p* = 0.04). Although only seen in 4 (10%) patients, common iliac node involvement was associated with a higher risk for progression (*p* = 0.007).

During the 24 months of primary follow-up there were three possibly treatment-related grade 3 toxicities including one case of gross hematuria in need of inpatient hospital care, one urethral fistula, and one case of recurring bladder obstruction requiring suprapubic catheter placement. Grade 2 toxicity was common, with 25/40 (62.5%) patients experiencing some treatment-related grade 2 toxicity during treatment or at some point during the 2‑year follow-up, with cystitis being the most frequently reported toxicity (Table 2).

**Table 2:** Treatment-related grade 2 toxicity as per CTCAE vs. 3.0

	Diarrhea (%)	Enteritis (%)	Proctitis (%)	Edema (%)	Cystitis (%)
Defining symptom	Increase of 4–6 stools per day over baseline; not interfering with ADL	Abdominal pain; mucus or blood in stool	Rectal discomfort	> 10–30% inter-limb discrepancy in volume or circumference	Frequency with dysuria; macroscopic hematuria
Treatment completion	11	0	0	0	22
Month 6	3	0	0	0	5
Month 12	3	0	0	0	9
Month 18	3	0	6	0	6
Month 24	0	6	0	0	6

*ADL* activities of daily living

After recovery from acute toxicity, quality of life as measured by the EORTC QLQ-C30 scores was not severely affected by the treatment on a long-term basis (supplementary Fig. 1).

## Discussion

Recently, large randomized controlled trials have been published to report on outcomes after whole-pelvic radiation therapy with or without androgen deprivation therapy in patients with a high risk for pelvic lymph node metastasis, most often assessed on the basis of the Roach formula [11–13]. In case of cN1-disease, however, mostly retrospective analyses have been published so far [14–17]. To our knowledge, PLATIN‑2 is the first prospective trial to report on long-term outcome after WPRT using IMRT with SIB to the nodal disease with neoadjuvant and concomitant ADT.

As part of a subanalysis of the STAMPEDE control arm, James et al. described a positive effect on failure-free survival (FFS; defined as lack of biochemical failure, systemic progression, and death from prostate cancer) in patients with high-risk prostate cancer including N+ disease when adding RT to ADT compared to ADT alone [18]. In their N+ M0 subcohort, FFS at 2 years was 89% in patients receiving RT and ADT with grade 3 genitourinary (GU) or gastrointestinal (GI) toxicities in about 5% of patients, which is similar to our results reported here.

In a retrospective analysis of 507 men treated with radical prostatectomy and lymph node dissection for node-positive prostate cancer, Boorjian et al. reported a superior biochemical recurrence-free survival after 10 years of 56%. Aside from the selection bias in a surgical cohort, these patients were not initially staged with node-positive disease and therefore represent a lower-risk cohort than the PLATIN‑2 cohort. Furthermore, lifelong androgen deprivation in 90% of patients as well as adjuvant or salvage radiation in 15% of patients have to be taken into account when comparing this outcome to the results reported here [19].

When compared to the long-term outcomes of the PLATIN-1 trial [6] with a high risk for but no evident nodal disease yet, both median OS and PFS are expectedly worse in PLATIN‑2 with macroscopic nodal involvement. Our relapse analysis shows that the radiation treatment in PLATIN‑2 provides excellent local control rates with tolerable toxicity. Progression was almost exclusively systemic and was less likely in patients who underwent a more sensitive pretherapeutic staging with PET/CT. Considering that most PET/CTs were conducted using a choline tracer instead of the more sensitive PSMA [20], advances in pretherapeutic staging may improve patient selection for local therapy, resulting in even better curation rates than those reported here. As seen in the postoperative PLATIN‑4 and PLATIN-5 trials, common iliac node involvement was a major risk factor for progression (unpublished data), whereas the number of involved nodes was not associated with a higher risk of progression.

Similar to the PLATIN-1 trial [6], no grade 3–4 GI toxicity was observed in our trial. In contrast to the PLATIN‑1 cohort, however, three grade 3 GU toxicities occurred. Revision of the radiation plan revealed no extraordinary dose distribution explaining the urethral fistula or bladder outlet obstruction, respectively. The gross hematuria was linked to an overdosage of phenprocoumon, although a radiogenic susceptibility for bleeding cannot be ruled out. Similar rates for grade 2+ GU and GI toxicities were reported in the whole-pelvic radiotherapy arm of the POP-RT trial [11], indicating a negligible additional risk for GU and GI toxicity by adding a SIB to the positive nodes. In the POP-RT trial, a more hypofractionated concept with 50 Gy in 25 fractions to the lymphatic drainage was safely applied in cN0 disease and was also similarly used with 46.8 Gy to the lymphatic drainage with SIB to the involved nodes up to 57.2 Gy in 26 fractions, with excellent infield control in the PLATIN-5 trial (unpublished data). Since then, even ultrahypofractionated concepts have been adopted with node-positive disease [21, 22].

Strengths of the study include the prospective nature, the long follow-up period, and the strict adherence to the study protocol. As adjuvant androgen deprivation was recommended although not an obligatory component of the protocol, treatment regimens varied inevitably depending on e.g. risk factors, toxicity, and the consulted urologist, potentially clouding PFS data during the first 24 months of follow-up. Limitations include the limited number of patients and the heterogenous staging modalities: since the study started patient recruitment in the pre-PSMA-PET/CT era, the missing PET/CT staging in patients recruited early to the trial may constitute a potential bias. Consistent use of PSMA-PET/CT as primary staging would probably have excluded the patients with early progression on MRI follow-up from enrolment. Subsequently, this study may have underestimated PFS and OS in patients with node-positive prostate cancer due to occult systemic metastases. Furthermore, in the context of high-risk prostate cancer and node-positive disease, the STAMPEDE trial has provided evidence supporting the use of intensified antiandrogen therapy with radiotherapy [23]. Specifically, the combination of ADT with abiraterone/prednisolone has demonstrated superior metastasis-free and overall survival compared to ADT alone and may be considered standard of care for patients with node-positive disease, even though the role of pelvic radiation in the setting of intensified antiandrogen is yet to be determined.

In summary, definitive radiation to the lymphatic drainage with a boost to the involved nodes and prostate is a safe and effective treatment option for node-positive prostate cancer. Considering the establishment of more sensitive pretherapeutic staging in high-risk prostate cancer with PSMA-PET/CT, more patients will be diagnosed in a nodal positive stage. For this growing patient cohort, the PLATIN-2 trial provides long-term data with excellent local control rates and tolerable toxicity.

### Supplementary Information

**Supplementary Fig. 1** QOL scores at baseline and 6‑, 12-, 18-, 24-month follow-up. The long-term data were collected after a median of 78 months.
