# Supplementary material for: Whole-pelvic irradiation with boost to involved nodes and prostate in node-positive prostate cancer—long-term data from the prospective PLATIN-2 trial
Source: Strahlenther Onkol. 2023 Aug 28;200(3):202–7. doi: 10.1007/s00066-023-02129-y (PMC10876493; doi:10.1007/s00066-023-02129-y)
Supplement: Supplementary file 1 — Supplementary Fig. 1 QOL scores at baseline and 6‑, 12-, 18-, 24-month follow-up. The long-term data were collected after a median of 78 months. [file 66_2023_2129_MOESM1_ESM.docx]

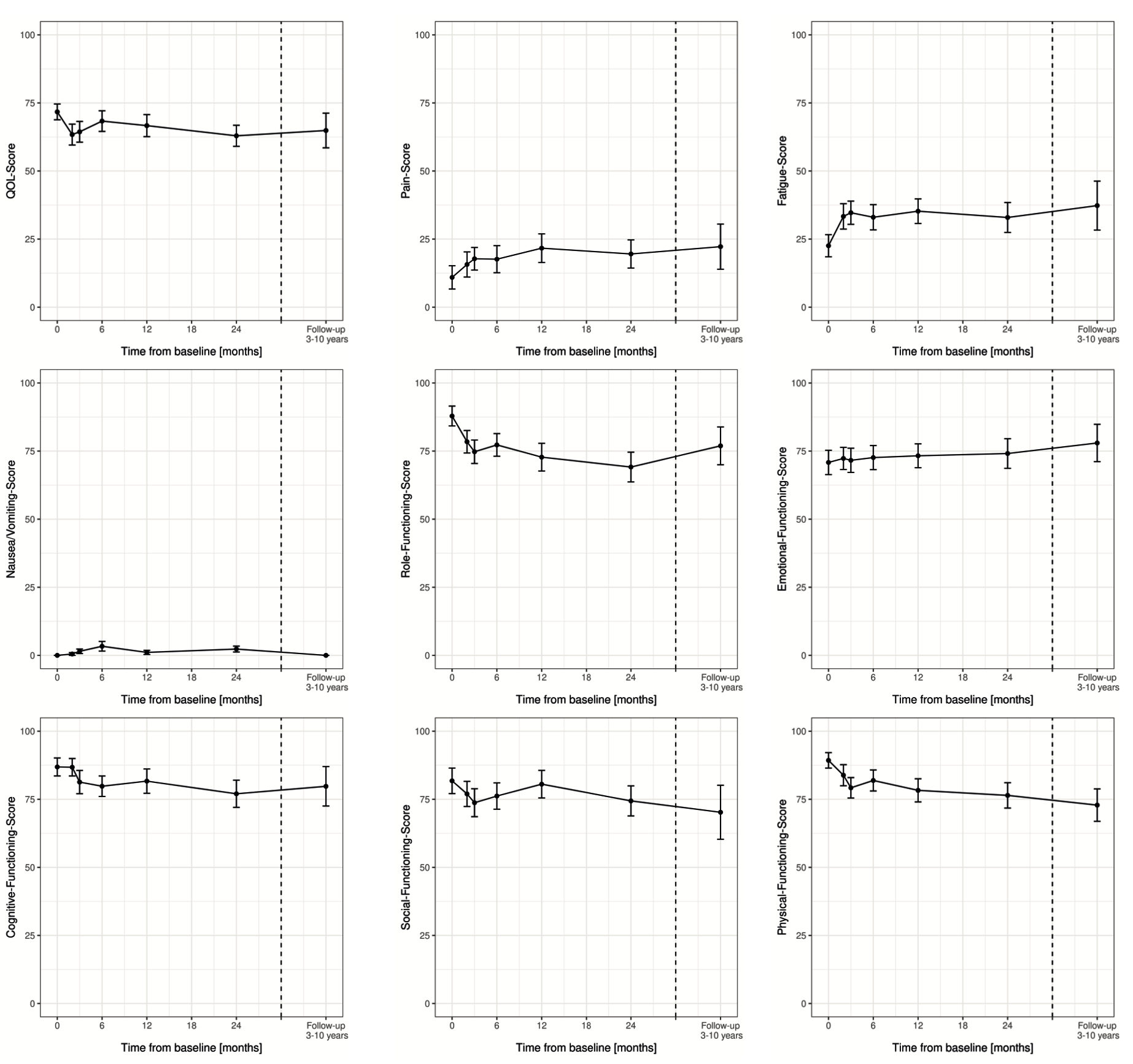


**Supplementary Fig. 1** QOL-Scores at baseline, 6, 12, 18, 24 months follow-up. The long-term data was collected after a median of 78 months.
